# 3-Methyl-4-oxo-2-phenyl-4*H*-chromene-8-carboxylic acid

**DOI:** 10.1107/S1600536808009732

**Published:** 2008-04-16

**Authors:** Zhi Hong, Hua-Jiang Jiang, Yu-Guo Zhuang, Hai-Chang Guo

**Affiliations:** a School of Pharmaceutical and Chemical Engineering, Taizhou University, Linhai 317000, People’s Republic of China

## Abstract

In the title compound, C_17_H_12_O_4_, the chromene unit is approximately planar, the maximum deviation from the mean plane being 0.0166 Å. The attached phenyl ring makes a dihedral angle of 53.2 (1)° with the fused ring system. The packing of the mol­ecules in the crystal structure is governed by C—H⋯O and O—H⋯O hydrogen-bonding inter­actions.

## Related literature

For related literature, see: Uneyama *et al.* (1985 ▶); Ghoneim *et al.* (2007 ▶); Da Re (1960 ▶, 1968 ▶); Sianesi (1972 ▶).
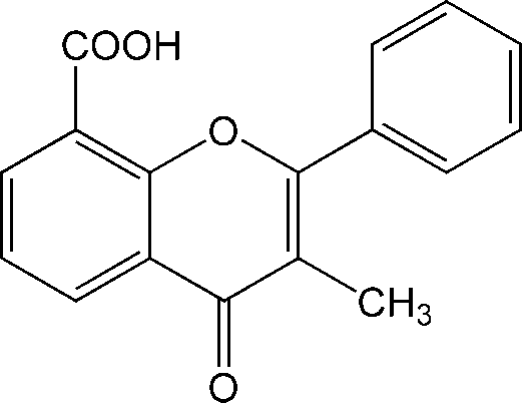


## Experimental

### 

#### Crystal data

C_17_H_12_O_4_
*M* *_r_* = 280.27
Triclinic, 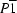

*a* = 7.2760 (8) Å
*b* = 9.6551 (10) Å
*c* = 11.3095 (12) Å
α = 65.965 (2)°
β = 79.748 (2)°
γ = 68.286 (2)°
*V* = 673.78 (12) Å^3^
*Z* = 2
Mo *K*α radiation
μ = 0.10 mm^−1^
*T* = 298 (2) K
0.35 × 0.27 × 0.18 mm

#### Data collection

Bruker APEX area-detector diffractometer
Absorption correction: multi-scan (*SADABS*; Bruker, 2002 ▶) *T*_min_ = 0.969, *T*_max_ = 0.989
3569 measured reflections
2354 independent reflections
2086 reflections with *I* > 2σ(*I*)
*R*_int_ = 0.015

#### Refinement

*R*[*F* ^2^ > 2σ(*F* ^2^)] = 0.058
*wR*(*F* ^2^) = 0.144
*S* = 1.10
2354 reflections
192 parameters
H-atom parameters constrained
Δρ_max_ = 0.20 e Å^−3^
Δρ_min_ = −0.25 e Å^−3^

### 

Data collection: *SMART* (Bruker, 2002 ▶); cell refinement: *SAINT* (Bruker, 2002 ▶); data reduction: *SAINT*; program(s) used to solve structure: *SHELXS97* (Sheldrick, 2008 ▶); program(s) used to refine structure: *SHELXL97* (Sheldrick, 2008 ▶); molecular graphics: *SHELXTL* (Sheldrick, 2008 ▶); software used to prepare material for publication: *SHELXL97*.

## Supplementary Material

Crystal structure: contains datablocks I, global. DOI: 10.1107/S1600536808009732/at2554sup1.cif

Structure factors: contains datablocks I. DOI: 10.1107/S1600536808009732/at2554Isup2.hkl

Additional supplementary materials:  crystallographic information; 3D view; checkCIF report

## Figures and Tables

**Table 1:** Hydrogen-bond geometry (Å, °)

*D*—H⋯*A*	*D*—H	H⋯*A*	*D*⋯*A*	*D*—H⋯*A*
O1—H1⋯O4^i^	0.82	1.86	2.615 (3)	154
C3—H3⋯O1	0.93	2.35	2.683 (3)	101
C14—H14⋯O2^ii^	0.93	2.57	3.347 (4)	141
C16—H16⋯O2^iii^	0.93	2.57	3.473 (5)	163

Symmetry codes: (i) 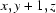; (ii) 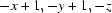; (iii) 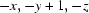.

**Fig. 1.**
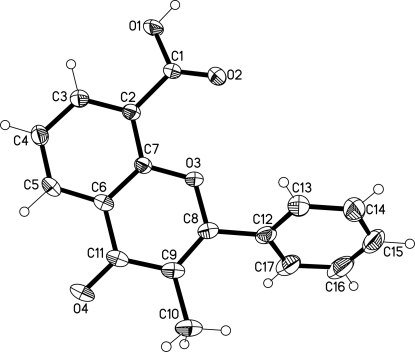
A view of the title compound, showing 30% probability ellipsoids for the non-hydrogen atoms and the atom-labelling scheme.

## supplementary crystallographic information

### Comment

In the title compound, 3-Methyl-4-oxo-2-phenyl-4*H*-chromene-8-carboxylic acid, is the key intermediate of flavoxate hydrochloride (Uneyama *et al.*, 1985). The flavoxate hydrochloride is a smooth muscle antispasmodic, especially on the urogenital tract (Ghoneim *et al.*, 2007). We report here the crystal structure of the title compound.

The 1-benzopyran unit is approximately planar, with a maximum deviation from the mean plane being 0.0166 A. The attached phenyl ring makes a dihedral angle of 53.2 (1)° with the fused ring system.The packing of the molecules in the crystal structure is mainly governed by C—H—Oπ hydrogen bonding interactions.

### Experimental

To a solution of 8-formyl-3-methyl-2-phenyl-4H-chromen-4-one (4 g, 15 mmol) in 2-butanone heated to 363–368 K, 31% of H_2_O_2_ (50 ml) was added four times at every 10 h intervals. After being stirred for 10 h, H_2_O_2_ was quenched with NaHSO_3_. The reaction mixture was acidified with 10% HCl and extracted with AcOEt. The extracts were concentrated in vacuo. The residue was dissolved in sat. NaHCO_3_ and extracted with AcOEt. The aqueous layer was acidified with 10% HCl and extracted with AcOEt. The extracts were dried with Na_2_SO_4_ and concentrated in vacuo. The residue was recrystallized from ethanol to give the title compound in a yield of 81%. m.p. 500.6–501.2 K. Crystals suitable for single-crystal X-ray diffraction were obtained by recrystallization from a mixed solvent of ethanol and dichloromethane (2:1 v/v) at room temperature.

### Refinement

All H atoms were placed geometrically at the distances of 0.93–0.96 Å for C—H and 0.826 Å for O—H and included in the refinement in riding motion approximation with U_iso_(H) = 1.2 or 1.5U_eq_ of the carrier atom.

### Crystal data

**Table:**

C_17_H_12_O_4_	*Z* = 2
*M**_r_* = 280.27	*F*_000_ = 292
Triclinic, *P*1	*D*_x_ = 1.381 Mg m^−^^3^
Hall symbol: -P 1	Mo *K*α radiation λ = 0.71073 Å
*a* = 7.2760 (8) Å	Cell parameters from 1477 reflections
*b* = 9.6551 (10) Å	θ = 2.5–27.7º
*c* = 11.3095 (12) Å	µ = 0.10 mm^−^^1^
α = 65.965 (2)º	*T* = 298 (2) K
β = 79.748 (2)º	Block, colourless
γ = 68.286 (2)º	0.35 × 0.27 × 0.18 mm
*V* = 673.78 (12) Å^3^	

### Data collection

**Table:**

Bruker APEX area-detector diffractometer	2354 independent reflections
Radiation source: fine-focus sealed tube	2086 reflections with *I* > 2σ(*I*)
Monochromator: graphite	*R*_int_ = 0.015
*T* = 298(2) K	θ_max_ = 25.0º
φ and ω scans	θ_min_ = 2.0º
Absorption correction: multi-scan(SADABS; Bruker, 2002)	*h* = −8→8
*T*_min_ = 0.969, *T*_max_ = 0.989	*k* = −11→11
3569 measured reflections	*l* = −13→12

### Refinement

**Table:**

Refinement on *F*^2^	Secondary atom site location: difference Fourier map
Least-squares matrix: full	Hydrogen site location: inferred from neighbouring sites
*R*[*F*^2^ > 2σ(*F*^2^)] = 0.058	H-atom parameters constrained
*wR*(*F*^2^) = 0.144	*w* = 1/[σ^2^(*F*_o_^2^) + (0.0631*P*)^2^ + 0.2396*P*] where *P* = (*F*_o_^2^ + 2*F*_c_^2^)/3
*S* = 1.10	(Δ/σ)_max_ = 0.004
2354 reflections	Δρ_max_ = 0.20 e Å^−^^3^
192 parameters	Δρ_min_ = −0.25 e Å^−^^3^
Primary atom site location: structure-invariant direct methods	Extinction correction: none

### Special details

**Table:**

Geometry. All e.s.d.'s (except the e.s.d. in the dihedral angle between two l.s. planes) are estimated using the full covariance matrix. The cell e.s.d.'s are taken into account individually in the estimation of e.s.d.'s in distances, angles and torsion angles; correlations between e.s.d.'s in cell parameters are only used when they are defined by crystal symmetry. An approximate (isotropic) treatment of cell e.s.d.'s is used for estimating e.s.d.'s involving l.s. planes.
Refinement. Refinement of *F*^2^ against ALL reflections. The weighted *R*-factor *wR* and goodness of fit *S* are based on *F*^2^, conventional *R*-factors *R* are based on *F*, with *F* set to zero for negative *F*^2^. The threshold expression of *F*^2^ > σ(*F*^2^) is used only for calculating *R*-factors(gt) *etc*. and is not relevant to the choice of reflections for refinement. *R*-factors based on *F*^2^ are statistically about twice as large as those based on *F*, and *R*-factors based on ALL data will be even larger.

### Fractional atomic coordinates and isotropic or equivalent isotropic displacement parameters (Å^2^)

**Table:**

	*x*	*y*	*z*	*U*_iso_*/*U*_eq_	
O1	0.2307 (3)	0.48247 (18)	0.52007 (15)	0.0620 (5)	
H1	0.2521	0.5681	0.4842	0.093*	
O2	0.3009 (3)	0.4730 (2)	0.32507 (15)	0.0690 (6)	
O3	0.2390 (2)	0.22602 (15)	0.30232 (13)	0.0398 (4)	
O4	0.2514 (3)	−0.23584 (17)	0.47596 (17)	0.0595 (5)	
C1	0.2644 (3)	0.4115 (2)	0.43715 (19)	0.0380 (5)	
C2	0.2526 (3)	0.2451 (2)	0.50288 (18)	0.0351 (5)	
C3	0.2552 (3)	0.1725 (2)	0.63616 (19)	0.0409 (5)	
H3	0.2558	0.2307	0.6844	0.049*	
C4	0.2568 (3)	0.0150 (2)	0.7004 (2)	0.0451 (5)	
H4	0.2597	−0.0311	0.7903	0.054*	
C5	0.2543 (3)	−0.0720 (2)	0.6318 (2)	0.0432 (5)	
H5	0.2562	−0.1776	0.6750	0.052*	
C6	0.2490 (3)	−0.0033 (2)	0.49658 (19)	0.0368 (5)	
C7	0.2471 (3)	0.1545 (2)	0.43312 (18)	0.0337 (4)	
C8	0.2394 (3)	0.1420 (2)	0.2298 (2)	0.0396 (5)	
C9	0.2489 (3)	−0.0139 (2)	0.2816 (2)	0.0443 (5)	
C10	0.2678 (4)	−0.1097 (3)	0.2009 (3)	0.0621 (7)	
H10A	0.1433	−0.1234	0.2021	0.093*	
H10B	0.3672	−0.2131	0.2357	0.093*	
H10C	0.3050	−0.0539	0.1134	0.093*	
C11	0.2487 (3)	−0.0952 (2)	0.4206 (2)	0.0422 (5)	
C12	0.2342 (3)	0.2459 (2)	0.0909 (2)	0.0466 (5)	
C13	0.3714 (4)	0.3244 (3)	0.0379 (2)	0.0570 (6)	
H13	0.4675	0.3111	0.0896	0.068*	
C14	0.3666 (5)	0.4226 (3)	−0.0916 (3)	0.0748 (8)	
H14	0.4607	0.4738	−0.1270	0.090*	
C15	0.2250 (6)	0.4448 (4)	−0.1677 (3)	0.0846 (10)	
H15	0.2233	0.5103	−0.2550	0.102*	
C16	0.0850 (6)	0.3712 (4)	−0.1163 (3)	0.0834 (10)	
H16	−0.0139	0.3893	−0.1681	0.100*	
C17	0.0903 (4)	0.2697 (3)	0.0127 (2)	0.0647 (7)	
H17	−0.0030	0.2176	0.0469	0.078*	

### Atomic displacement parameters (Å^2^)

**Table:**

	*U*^11^	*U*^22^	*U*^33^	*U*^12^	*U*^13^	*U*^23^
O1	0.1144 (15)	0.0418 (9)	0.0473 (9)	−0.0423 (10)	0.0084 (9)	−0.0233 (7)
O2	0.1327 (17)	0.0529 (10)	0.0399 (9)	−0.0575 (11)	0.0093 (9)	−0.0161 (8)
O3	0.0565 (9)	0.0317 (7)	0.0375 (8)	−0.0167 (6)	−0.0028 (6)	−0.0168 (6)
O4	0.0792 (12)	0.0333 (8)	0.0753 (12)	−0.0259 (8)	−0.0028 (9)	−0.0224 (8)
C1	0.0499 (12)	0.0313 (10)	0.0385 (12)	−0.0178 (9)	−0.0023 (9)	−0.0141 (9)
C2	0.0380 (11)	0.0307 (10)	0.0391 (11)	−0.0123 (8)	0.0004 (8)	−0.0153 (8)
C3	0.0480 (12)	0.0370 (11)	0.0402 (12)	−0.0140 (9)	−0.0002 (9)	−0.0174 (9)
C4	0.0543 (13)	0.0380 (11)	0.0372 (11)	−0.0164 (10)	0.0012 (9)	−0.0087 (9)
C5	0.0482 (12)	0.0269 (10)	0.0497 (13)	−0.0150 (9)	0.0008 (9)	−0.0086 (9)
C6	0.0342 (10)	0.0296 (10)	0.0494 (12)	−0.0119 (8)	0.0000 (8)	−0.0169 (9)
C7	0.0355 (10)	0.0298 (9)	0.0364 (11)	−0.0115 (8)	0.0009 (8)	−0.0133 (8)
C8	0.0399 (11)	0.0419 (11)	0.0452 (12)	−0.0128 (9)	−0.0004 (9)	−0.0250 (9)
C9	0.0420 (12)	0.0424 (11)	0.0598 (14)	−0.0149 (9)	0.0019 (10)	−0.0307 (10)
C10	0.0734 (17)	0.0547 (14)	0.0766 (18)	−0.0214 (13)	0.0020 (13)	−0.0439 (13)
C11	0.0400 (11)	0.0326 (10)	0.0600 (14)	−0.0138 (9)	0.0007 (9)	−0.0224 (10)
C12	0.0565 (13)	0.0405 (11)	0.0449 (12)	−0.0065 (10)	−0.0022 (10)	−0.0262 (10)
C13	0.0740 (17)	0.0481 (13)	0.0502 (14)	−0.0209 (12)	0.0011 (12)	−0.0203 (11)
C14	0.113 (2)	0.0549 (15)	0.0545 (16)	−0.0303 (16)	0.0168 (16)	−0.0239 (13)
C15	0.142 (3)	0.0570 (17)	0.0416 (15)	−0.0107 (19)	−0.0024 (18)	−0.0259 (13)
C16	0.110 (3)	0.078 (2)	0.0595 (18)	0.0011 (18)	−0.0324 (17)	−0.0400 (16)
C17	0.0720 (17)	0.0719 (17)	0.0586 (16)	−0.0143 (14)	−0.0126 (13)	−0.0368 (13)

### Geometric parameters (Å, °)

**Table:**

O1—C1	1.314 (2)	C8—C12	1.478 (3)
O1—H1	0.8200	C9—C11	1.443 (3)
O2—C1	1.189 (2)	C9—C10	1.502 (3)
O3—C7	1.355 (2)	C10—H10A	0.9600
O3—C8	1.367 (2)	C10—H10B	0.9600
O4—C11	1.236 (2)	C10—H10C	0.9600
C1—C2	1.499 (3)	C12—C13	1.381 (3)
C2—C3	1.380 (3)	C12—C17	1.385 (3)
C2—C7	1.410 (3)	C13—C14	1.381 (4)
C3—C4	1.390 (3)	C13—H13	0.9300
C3—H3	0.9300	C14—C15	1.360 (5)
C4—C5	1.362 (3)	C14—H14	0.9300
C4—H4	0.9300	C15—C16	1.366 (5)
C5—C6	1.398 (3)	C15—H15	0.9300
C5—H5	0.9300	C16—C17	1.385 (4)
C6—C7	1.391 (3)	C16—H16	0.9300
C6—C11	1.466 (3)	C17—H17	0.9300
C8—C9	1.354 (3)		
			
C1—O1—H1	109.5	C11—C9—C10	117.80 (19)
C7—O3—C8	120.38 (15)	C9—C10—H10A	109.5
O2—C1—O1	123.45 (17)	C9—C10—H10B	109.5
O2—C1—C2	125.38 (18)	H10A—C10—H10B	109.5
O1—C1—C2	111.17 (17)	C9—C10—H10C	109.5
C3—C2—C7	117.48 (17)	H10A—C10—H10C	109.5
C3—C2—C1	120.09 (17)	H10B—C10—H10C	109.5
C7—C2—C1	122.37 (17)	O4—C11—C9	123.31 (19)
C2—C3—C4	121.87 (19)	O4—C11—C6	120.0 (2)
C2—C3—H3	119.1	C9—C11—C6	116.63 (17)
C4—C3—H3	119.1	C13—C12—C17	118.9 (2)
C5—C4—C3	120.06 (19)	C13—C12—C8	119.9 (2)
C5—C4—H4	120.0	C17—C12—C8	121.1 (2)
C3—C4—H4	120.0	C12—C13—C14	120.3 (3)
C4—C5—C6	120.32 (17)	C12—C13—H13	119.9
C4—C5—H5	119.8	C14—C13—H13	119.9
C6—C5—H5	119.8	C15—C14—C13	120.3 (3)
C7—C6—C5	119.23 (17)	C15—C14—H14	119.8
C7—C6—C11	119.48 (18)	C13—C14—H14	119.8
C5—C6—C11	121.28 (17)	C14—C15—C16	120.2 (3)
O3—C7—C6	120.94 (17)	C14—C15—H15	119.9
O3—C7—C2	118.04 (16)	C16—C15—H15	119.9
C6—C7—C2	121.02 (18)	C15—C16—C17	120.1 (3)
C9—C8—O3	123.39 (19)	C15—C16—H16	119.9
C9—C8—C12	127.06 (18)	C17—C16—H16	119.9
O3—C8—C12	109.53 (16)	C12—C17—C16	120.1 (3)
C8—C9—C11	119.07 (18)	C12—C17—H17	120.0
C8—C9—C10	123.1 (2)	C16—C17—H17	120.0
			
O2—C1—C2—C3	164.8 (2)	C12—C8—C9—C11	−178.8 (2)
O1—C1—C2—C3	−14.5 (3)	O3—C8—C9—C10	−174.3 (2)
O2—C1—C2—C7	−12.4 (3)	C12—C8—C9—C10	3.7 (4)
O1—C1—C2—C7	168.23 (18)	C8—C9—C11—O4	177.9 (2)
C7—C2—C3—C4	1.4 (3)	C10—C9—C11—O4	−4.4 (3)
C1—C2—C3—C4	−176.0 (2)	C8—C9—C11—C6	−3.3 (3)
C2—C3—C4—C5	−0.5 (3)	C10—C9—C11—C6	174.40 (19)
C3—C4—C5—C6	−0.3 (3)	C7—C6—C11—O4	179.63 (19)
C4—C5—C6—C7	0.2 (3)	C5—C6—C11—O4	0.6 (3)
C4—C5—C6—C11	179.2 (2)	C7—C6—C11—C9	0.8 (3)
C8—O3—C7—C6	−2.2 (3)	C5—C6—C11—C9	−178.21 (18)
C8—O3—C7—C2	178.03 (17)	C9—C8—C12—C13	−125.6 (2)
C5—C6—C7—O3	−179.03 (17)	O3—C8—C12—C13	52.7 (3)
C11—C6—C7—O3	1.9 (3)	C9—C8—C12—C17	56.1 (3)
C5—C6—C7—C2	0.7 (3)	O3—C8—C12—C17	−125.6 (2)
C11—C6—C7—C2	−178.32 (17)	C17—C12—C13—C14	−1.2 (3)
C3—C2—C7—O3	178.24 (17)	C8—C12—C13—C14	−179.5 (2)
C1—C2—C7—O3	−4.5 (3)	C12—C13—C14—C15	1.0 (4)
C3—C2—C7—C6	−1.5 (3)	C13—C14—C15—C16	0.6 (4)
C1—C2—C7—C6	175.80 (18)	C14—C15—C16—C17	−1.9 (4)
C7—O3—C8—C9	−0.4 (3)	C13—C12—C17—C16	−0.1 (3)
C7—O3—C8—C12	−178.76 (16)	C8—C12—C17—C16	178.2 (2)
O3—C8—C9—C11	3.2 (3)	C15—C16—C17—C12	1.7 (4)

### Hydrogen-bond geometry (Å, °)

**Table:**

*D*—H···*A*	*D*—H	H···*A*	*D*···*A*	*D*—H···*A*
O1—H1···O4^i^	0.82	1.86	2.615 (3)	154
C3—H3···O1	0.93	2.35	2.683 (3)	101
C14—H14···O2^ii^	0.93	2.57	3.347 (4)	141
C16—H16···O2^iii^	0.93	2.57	3.473 (5)	163

Symmetry codes: (i) *x*, *y*+1, *z*; (ii) −*x*+1, −*y*+1, −*z*; (iii) −*x*, −*y*+1, −*z*.
